# Phylogeny of Cutthroat Trout (*Oncorhynchus clarkii*) Based on Full Mitochondrial Genomes With Comments on Species Delimitation and Taxonomy

**DOI:** 10.1111/eva.70304

**Published:** 2026-07-15

**Authors:** Tanner S. Van Orden, Kevin B. Rogers, Peter C. Searle, Andrea L. Kokkonen, Dennis K. Shiozawa, Richard Paul Evans

**Affiliations:** ^1^ Department of Fisheries University of Alaska Fairbanks Alaska USA; ^2^ Aquatic Research Section Colorado Parks and Wildlife Steamboat Springs Colorado USA; ^3^ Department of Ecology and Evolutionary Biology Cornell University Ithaca New York USA; ^4^ Department of Microbiology and Molecular Biology Brigham Young University Provo Utah USA; ^5^ Department of Biology Brigham Young University Provo Utah USA

## Abstract

Despite the important role that species concepts and taxonomy play in understanding the evolution of species, taxonomists have struggled to describe the relationship between many organisms. Here, using Cutthroat Trout as a case study, we illustrate how full mitochondrial genomes and broad taxonomic sampling can provide further insight on disputed taxonomic relationships, and aid in management and conservation decisions of different species. Inconsistencies in the taxonomy of Cutthroat Trout inspired a special workshop at the 2015 annual meeting of the American Fisheries Society that emphasized the need for revision. To further resolve the relationship between different Cutthroat Trout groups, we sequenced and assembled full mitochondrial genomes from 123 Cutthroat Trout from across their native range. We used maximum likelihood and Bayesian phylogenetic approaches to examine the evolutionary relationships between all named Cutthroat Trout subspecies. Through these analyses, we find eight lineages of Cutthroat Trout that diverged more than one million years ago, and at least 12 lineages that diverged more than 790 thousand years ago, highlighting that despite the ancient split with Rainbow Trout at around ten million years ago, the modern diversity seen within the Cutthroat Trout Species Complex is much more recent. We do not find that Coastal, Westslope, or Lahontan Cutthroat Trout are substantially more different than the lineages in the Yellowstone complex. As such, we continue to recognize a single Cutthroat Trout species ( *Oncorhynchus clarkii* ) comprised of eleven distinct subspecies level clades, maintaining consistency with ongoing conservation efforts, management plans and ESA‐related decisions, while also preserving stability associated with historical nomenclature. Overall, we highlight that using full mitochondrial genomes can increase phylogenetically informative information and that the consistent nature of mitochondrial genomes across the tree of life allows for a valuable and measurable comparison of different species concepts in closely related taxa.

## 1 Introduction

Species concepts and taxonomy have always been an essential part of understanding the evolution of organisms because they provide a framework for recognizing and defining distinct groups (Wilson 1992). Additionally, species concepts play an important role in the protection and conservation of species, yet what defines a species remains elusive (Mayden 1997). Over the last 80 years, at least 34 concepts have been proposed, but no consensus on which species concept is the most accurate or useful has been achieved (Mayr 1968; Mayden 1999; Frankham et al. 2012; Zachos 2018). While new concepts continue to proliferate (Hill 2017; Shanker et al. 2017; Zachos 2018), most tend to move away from Mayr's biological species concept, which uses reproductive isolation to delineate species. Instead, newer approaches acknowledge even finer diversity. For example, the phylogenetic species concept allows any monophyletic group to be treated as a species (Nixon and Wheeler 1990), while the Unified Species Concept defines species as ‘separately evolving metapopulation lineages’ (De Queiroz 2007). While this transition helps highlight complex taxonomic relationships, it at times has resulted in the rapid inflation of described species within well studied taxonomic groups and shaken stability in nomenclature—conflicting with the stated objectives of the International Commission on Zoological Nomenclature and the American Fisheries Society (Page et al. 2023).

Different morphological and genetic markers represent distinct lines of evidence for delineating species (De Queiroz 2007). Of these genetic markers, mitochondrial DNA (mtDNA) has been used extensively in both population genetics and systematics (Desalle et al. 2007). This is because mtDNA has several practical advantages in phylogenetic analyses over nuclear DNA. For instance, mitochondrial genes evolve faster on average than nuclear genes in vertebrates due to higher mutation rates. Additionally, mtDNA's smaller effective population sizes lead to faster coalescent times (Allio et al. 2017), making them useful for resolving relationships in recently diverged clades (e.g., species and subspecies level) (Finnegan et al. 2025; Xing et al. 2025). Further, rates of evolution vary in mitochondrial genes which means some genes are better at resolving shallower divergence because they evolve faster (protein‐coding genes), while other genes can resolve deeper divergence because they are more conserved (tRNA, rRNA) (Mueller 2006; Finnegan et al. 2025). Furthermore, due to its small genome size and simple structure, mtDNA can be easily sequenced and assembled into mitochondrial genomes (henceforth mitogenomes; Desalle et al. 2007). Mitogenomes can also broaden the taxonomic scope of a study because these markers can be gathered and assembled from previous sequencing datasets not initially designed for mitochondrial genome assembly (Miller and Manica 2021). Importantly, mitochondrial genes and genomes can be recovered from degraded samples, making them particularly useful for studies using historical specimens (Metcalf et al. 2012; Templeton et al. 2013; Kehlmaier et al. 2019; Raxworthy and Smith 2021).

Despite these advantages, there are also challenges when inferring phylogenies strictly with mtDNA, a topic that has been debated extensively (Shaw 2002; Lin and Danforth 2004; Rubinoff and Holland 2005). As a single, maternally inherited locus, mitogenomes only represent one gene tree, limiting the taxonomic conclusions that can be drawn from these data. Further, saturation, mitochondrial capture, positive selection, the presence of nuclear mitochondrial pseudogenes, hybridization and introgression can all contribute to discordance between mitochondrial and nuclear phylogenies (Rubinoff and Holland 2005; Perea et al. 2016; Kang and Hu 2023; Thalmann et al. 2004). Numerous studies across diverse animal groups have documented mito‐nuclear discordance (Funk and Omland 2003; Toews and Brelsford 2012; Good et al. 2015; Dallaire et al. 2025; Crespi and Fulton 2004; Englmaier et al. 2024), highlighting that findings drawn from mtDNA should ultimately be corroborated with nuclear genetic data when evaluating taxonomic relationships. Despite these limitations, mtDNA continues to provide important insight on the relationship between species across broad population and taxonomic levels.

To illustrate the utility of using mitogenomes for species delimitation, we explore the relationships in a taxonomically disputed group, the Cutthroat Trout ( *Oncorhynchus clarkii* ). Cutthroat Trout are a Pacific trout species native to the West Coast of North America from Alaska south to California, and inland through the Columbia and upper Missouri river basins, the Great Basin (including the Lahontan and Bonneville basins), and along with the headwaters of the Colorado, Rio Grande, South Platte and Arkansas river basins. Long‐term isolation in these discrete geographic regions has led to the evolution of many distinct lineages of Cutthroat Trout, and as such, Cutthroat Trout have been classified into many different taxonomic groupings (Behnke 1992, 2002; Trotter 2008). Taxonomists have struggled to describe the diversity found within Cutthroat Trout, with up to 40 separate species being recognized before many were synonymized as either subspecies of Rainbow Trout (formerly *Salmo gairdnerii* ) or Cutthroat Trout (formerly *Salmo clarkii* ) (Jordan et al. 1930; Miller 1950). More recently, various authors have recognized between 11 and 16 different subspecies of Cutthroat Trout within this native range (Smith and Stearley 1989; Behnke 1992, 2002; Houston et al. 2012; Penaluna et al. 2016; Budy et al. 2019; Kokkonen et al. 2024).

Recent studies revealed inconsistencies in the taxonomy of Cutthroat Trout at the subspecies level (Shiozawa and Williams 1992; Metcalf et al. 2007, 2012; Loxterman and Keeley 2012; Bestgen et al. 2019). This precipitated a special workshop at the 2015 annual meeting of the American Fisheries Society that emphasized the need for a revised taxonomy of Cutthroat Trout. Workshop participants settled on the Unified Species Concept (*sensu* De Queiroz 2007) as a way to delineate diversity in Cutthroat Trout, as relationships between them continue to be resolved. This workshop aimed to identify lineages across all subspecies that were on separate evolutionary trajectories, which they referred to as uniquely identifiable evolutionary units (UIEUs). After much deliberation, at least 25 UIEUs were recognized across the range of Cutthroat Trout (Trotter et al. 2018). However, in November 2023, the Joint Committee of the American Fisheries Society and the American Society of Ichthyologists and Herpetologists moved to reclassify Cutthroat Trout into four species without any named subspecies. These four species include Coastal Cutthroat Trout ( *Oncorhynchus clarkii* ), Lahontan Cutthroat Trout (*Oncorhynchus henshawi*), Westslope Cutthroat Trout (*Oncorhynchus lewisi*) and Rocky Mountain Cutthroat Trout (*Oncorhynchus virginalis*) (Page et al. 2023). This reclassification was described by Markle (2018) who proposed an ‘interim classification’ of Cutthroat Trout before more work could be done to allow a systematic reclassification. However, this does not adequately reflect the diversity described in that same workshop (Campbell et al. 2018; Rogers, Bestgen, et al. 2018; Shiozawa et al. 2018), or from other taxonomic studies (Metcalf et al. 2007, 2012; Pritchard et al. 2009; Houston et al. 2012; Loxterman and Keeley 2012; Bestgen et al. 2019; Kokkonen et al. 2024) that reveal substantial genetic differentiation between the remaining Cutthroat Trout subspecies and lineages, that appear as divergent as the four new ‘species’. By elevating select subspecies above others, this decision failed to resolve confusion surrounding the Cutthroat Trout subspecies and raised additional questions about why former Cutthroat trout subspecies should be ranked on par with widely recognized species (e.g., Rainbow Trout).

To provide increased resolution and to clarify polytomies that arose in earlier single mitochondrial gene studies, we sequenced and inferred phylogenies using full mitogenomes from Cutthroat Trout across their native range. Although mitogenomes represent only one line of evidence for species delimitation, they were particularly useful for this study because they allowed us to compare relationships across a broader taxonomic range than previous studies, including an extinct subspecies, the Yellowfin Cutthroat Trout ( *Oncorhynchus clarkii macdonaldi* ). Additionally, their recent divergence, as well as geographic isolation and availability of historical museum specimens argue for mitochondrial phylogenetics as a logical asset to assist with resolving inadequacies in native trout taxonomy. Here we use maximum likelihood and Bayesian phylogenetic approaches with mitogenomic data to clarify evolutionary relationships across the entire Cutthroat Trout species complex, which in turn can serve as a model for other recently diverged taxa with complicated evolutionary histories.

## 2 Methods

### 2.1 Mitochondrial Genome Acquisition

Samples used in the project were collected by the authors or state and federal fisheries biologists between 1990 and 2022. Isolated DNA and fin clips were housed at the M. L. Bean Life Science Museum (MLBM, Provo, UT; Appendix S1), or at Pisces Molecular (Boulder, Colorado; Appendix S1). These samples were chosen based on prior knowledge of mitochondrial haplotype distribution to reflect mitochondrial haplotype diversity instead of random geographic sampling. This was because our primary goal was to focus on genetic divergence between clades rather than contemporary geographic position which would conflate stocking driven distribution with natural evolutionary history. Sequence data for this project was generated across multiple independent projects at both Brigham Young University and Pisces Molecular. Overall, DNA sequencing runs were of varying depths, and mitogenomes were assembled using both reference‐guided and *de novo* methods.

### 2.2 Bean Life Science Museum Sequencing and Mitochondrial Genome Assembly

Whole genomic DNA was isolated using DNeasy tissue kits (Qiagen, Germantown, MD, USA) following the manufacturer's recommended protocol. DNA purity and quantity was verified using a NanoDrop One (Thermo Fisher Scientific, Waltham, MA, USA) and a Qubit fluorometer with a dsDNA Broad Range assay kit. Isolated DNA from 85 MLBM samples were sent to Novogene America (Davis, California, USA). Libraries were prepared with an Illumina Nextera DNA Flex kit and sequenced on an Illumina Hi‐Seq 2500 (Illumina, San Diego, CA, USA) using paired‐end reads (2 × 150 bp) (Appendix S1). DNA libraries were randomly distributed across 4 lanes, and 2 GB of paired‐end reads were sequenced for each sample. Five DNA samples were sent to the University of Oregon Sequencing Centre for library preparation and sequencing (Appendix S1). Each library was verified using a MiSeq Nano and then sequenced on an Illumina NovaSeq—S4—PE 150 Cycle (Illumina, San Diego, CA, USA) using paired‐end reads (2 × 150 bp) for a minimum of 33 million paired‐end reads per sample (16.5 million reads per direction). Raw sequence reads from MLBM samples were assembled into full mitogenomes using MitoZ v3.6 (Meng et al. 2019) which trims out adapters and low‐quality reads using fastp (Chen et al. 2018) and assembles mitogenomes using MegHit v1.2.9 (Li et al. 2015).

### 2.3 Pisces Molecular Sequencing and Mitochondrial Genome Assembly

Total genomic DNA was isolated using DNeasy tissue kits (Qiagen, Germantown, MD, USA) using the manufacturers' recommended ‘mouse tail’ protocol. Total mitochondrial DNA was amplified from each sample in three fragments, using primer pairs as follows: Fragment 1 (forward primer in tRNA‐Glx, reverse primer in ND3, 5975 bp), Fragment 2 (forward primer in tRNA‐Gly, reverse primer in CytB, 5972 bp) and Fragment 3 (forward primer in CytB, reverse primer in ND2, 5635 bp). Three amplified fragments from 32 samples were purified with Qiaquick PCR purification columns (Qiagen, Germantown, MD, USA) and sent to the University of Colorado BioFrontiers Institute for library preparation and sequencing (Appendix S1). Libraries were prepared by tagmentation and indexed using an Illumina Nextera XT DNA Library Preparation kit. Libraries were sequenced with an Illumina MiSeq (Illumina, San Diego, CA, USA) using paired end reads (2 × 150 bp). Raw reads were processed to trim adapter and primer sequences then assembled into a full mitochondrial genome using Geneious R6.1 (Biomatters, Auckland, NZ).

### 2.4 Yellowfin Cutthroat Trout Mitochondrial Genome Sequence and Assembly

The Smithsonian National Museum of National History (NMNH) collection includes five Yellowfin Cutthroat Trout specimens (one holotype; four paratypes) collected by Jordan Star in 1889. Metcalf et al. (2012) used four of these samples and were successfully able to isolate and amplify DNA from each specimen, but since the study relied on PCR and Sanger sequencing, only several short mitochondrial fragments were obtained. To evaluate whether modern whole genome shotgun sequencing could be used to recover whole mitochondrial genomes from historic samples, we analyzed the fifth previously unsampled individual using mitochondrial genome enrichment, Illumina sequencing and reference‐guided assembly. Specifically, total genomic DNA was isolated using DNeasy tissue kits (Qiagen, Germantown, MD, USA) using the manufacturers' recommended ‘mouse tail’ protocol. Total mitochondrial DNA was amplified in three overlapping fragments as described above and then purified using the Qiaquick PCR purification columns (Qiagen, Germantown, MD, USA) before being sent to the University of Colorado BioFrontiers Institute for library preparation and sequencing in a facility that had not previously been used for Cutthroat Trout samples (CU Cooperative Institute for Research in Environmental Sciences building). Its library was prepared by tagmentation and indexed using an Illumina Nextera XT DNA Library Preparation kit. This library was sequenced with an Illumina MiSeq (Illumina, San Diego, CA, USA) using paired end reads (2 × 150 bp). Short sequence reads were processed to trim adapter and primer sequences then assembled into a full mitochondrial genome using Geneious R6.1 (Biomatters, Auckland, NZ).

### 2.5 Mitogenome Annotation and Sequence Comparison

Each mitogenome was annotated using MitoFish v4.09 (Iwasaki et al. 2013; Sato et al. 2018; Zhu et al. 2023). We incorporated publicly available Cutthroat Trout (*Oncorhynchus clarkii*), Rainbow Trout ( *Oncorhynchus mykiss* ) and Chinook Salmon ( *Oncorhynchus tshawytscha* ) mitogenomes in all downstream analyses (Brown et al. 2006; Dziedzic et al. 2023; Westfall et al. 2023; Diver et al. 2024). Rainbow Trout are the sister species to Cutthroat Trout (Smith and Stearley 1989) and provide context to the branch lengths found between Cutthroat Trout subspecies and lineages. Chinook Salmon represent a robust outgroup to root the trees as they are closely related to both Pacific trout species (Behnke 2002; Wilson and Turner 2009; Brunelli et al. 2013). All genes were aligned using Mafft v7.525 (Katoh and Standley 2013), and a concatenated sequence super matrix was created using FASconCAT v1.7 (Kück and Longo 2014). We used MEGA v12 to calculate average pairwise percent differences between each sample in this concatenated sequence super matrix (Kumar et al. 2024) so that the genetic distance between Chinook Salmon, Rainbow Trout and all Cutthroat Trout lineages could be estimated.

### 2.6 Phylogenetic Analyses

ModelFinder in IQtree v2.3.4 was run twice to find the best model of sequence evolution for each gene: once to test all models of sequence evolution available in IQtree v2.3.4, and again to test all models of sequence evolution available in BEAST v2.6 (Minh et al. 2020). IQtree v2.3.4 was used to generate a maximum likelihood phylogeny using 1000 ultrafast bootstrap alignments and 1000 iterations (Minh et al. 2020). A molecular clock and topology search were conducted using BEAST v2.6 with the calibrated Yule process of speciation with a chain length of 240,000,000 (Bouckaert et al. 2014). Given the computational demands of running a Bayesian dating analysis with 148 taxa, this analysis was exclusively conducted with an optimized relaxed clock which allows variation in mutation rates between branches while running up to 65 times faster than other relaxed clocks implemented in BEAST (Douglas et al. 2021). We calibrated this molecular clock using an *Oncorhynchus belli* fossil, setting the most recent common ancestor of Rainbow Trout and Cutthroat Trout lineages as a uniform distribution between 9.7 and 10.7 Ma (Stearley and Smith 2016). A uniform distribution prior was chosen as the *Oncorhynchus belli* fossil provided a minimum most recent common ancestor constraint but gives no additional information on a particular date within that window. All Bayesian Trees were annotated with TreeAnnotator v2.7.6 using common ancestor heights and a burn‐in percentage of 10% (Drummond and Rambaut 2007). Tracer v1.7.2 was used to assess adequate chain mixing by ensuring that all estimated sample sizes (ESS) were > 200 for each parameter (Rambaut et al. 2018).

## 3 Results

Overall, the varying sequencing techniques and assembly methods we used yielded complete mitogenomes for 117 out of 123 samples sequenced for this study. These mitogenomes consisted of 13 protein‐coding genes, two rRNA genes, and 22 tRNA genes ranging in length from 16,655 to 16,661 bp (Appendix S1). All but 6 of the 90 mitogenomes from the Bean Life Science Museum, were fully annotated (no missing data). Five mitogenomes had fragmented genes with missing data ranging from 8 to 50 bp. One sample had 492 bp of missing data (Appendix S1). These mitogenomes were still included in subsequent analyses as missing data was minimal. Coverage for these mitogenomes ranged from 17.4× to 404.2× with an average of 58.5× (Appendix S1). All 33 samples from Pisces Molecular were fully annotated and had no missing data. Coverage ranged from 14.1× to 17,795× with an average of 9342× (Appendix S1). Annotated mitogenomes were submitted to GenBank under BioProject accession number PRJNA1301035.

Both the maximum likelihood and Bayesian phylogenies identified the same most likely topology and arranged all Cutthroat Trout mitogenomes into twelve well defined mitochondrial haplogroups (Figure 2). These groups coincided with previously identified subspecies and lineages that included 6 Coastal Cutthroat Trout (*O. c. clarkii*), 14 Lahontan Cutthroat Trout (*O. c. henshawi*), 9 Westslope Cutthroat Trout (*O. c. lewisi*), 14 Yellowstone Cutthroat Trout (*O. c. bouvieri*), 9 Bear River Cutthroat Trout (undescribed lineage of Yellowstone Cutthroat Trout; _BR_YCT), 19 Bonneville Cutthroat Trout (*O. c. utah*), 18 Green River Cutthroat Trout (undescribed lineage of Colorado River Cutthroat Trout; *O. c. pleuriticus*; _GR_CRCT), 15 Rio Grande Cutthroat Trout (*O. c. virginalis*), 1 Yellowfin Cutthroat Trout (*O. c. macdonaldi*), 5 Greenback Cutthroat Trout (*O. c. ssp*; see Rogers, Bestgen, et al. 2018; Rogers, White, and Japhet 2018), 5 San Juan Cutthroat Trout (undescribed lineage of CRCT; _SJ_CRCT), and 17 Uncompahgre Cutthroat Trout (undescribed lineage of CRCT; _UP_CRCT). Groups in the maximum likelihood phylogeny had bootstrap support values ranging from 77 to 100 (Figure 2 and Figure S1). Groups in the Bayesian phylogeny had posterior probabilities ranging from 0.66 to 1.0, though only two nodes had support < 0.99 (Figure 2 and Figure S1). Additionally, the Bayesian Clock showed that although Cutthroat Trout and Rainbow Trout diverged ~10.2 Ma, Coastal Cutthroat Trout did not diverge from other Cutthroat Trout lineages until > 2 Ma (Figure 2). Our molecular clock identified at least 8 lineages of Cutthroat Trout that diverged > 1 Ma and 12 lineages of Cutthroat Trout that diverged > 790 ka (Figure 2).

The pairwise distance between all Cutthroat Trout lineages using the full mitogenomes was similar, with an average distance of 1.30% (Table 2). Notably, the _BR_YCT and Yellowstone Cutthroat Trout had the smallest pairwise distance at 0.3%, while the largest sequence divergence was present between Yellowfin Cutthroat Trout and Coastal Cutthroat Trout at 2.00%. The greatest pairwise distance observed in this entire sample set was between Chinook Salmon and the Yellowfin Cutthroat Trout at 7.20%, while all combined Rainbow Trout and Cutthroat Trout lineages differed by 4.98%.

Genetic differences between Cutthroat Trout haplogroups were highly variable depending on which specific genes were investigated. On average, protein‐coding genes were more variable than tRNA and rRNA. In terms of protein‐coding genes, ATPase8 was the least variable with 0.87% average pairwise distance and ND2 was the most variable with an average pairwise distance of 2.04% (Table 1). As for tRNA genes, tRNA‐Arg, tRNA‐Met and tRNA‐Asn were completely conserved across all Cutthroat Trout haplogroups, while tRNA‐Trp was the most variable with an average pairwise distance of 1.91%. Both rRNA genes were very conserved across all Cutthroat Trout haplogroups. The 12S subunit had an average pairwise distance of 0.43% and the 16S subunit had an average pairwise distance of 0.51%. While tRNA‐Arg and tRNA‐Met were completely conserved across all Cutthroat Trout haplogroups, they still had one and three parsimony informative sites, respectively, between Rainbow Trout and Cutthroat Trout.

**TABLE 1:** Location, length, average pairwise difference, and other supporting measures for all protein‐coding genes for all Cutthroat Trout haplogroups.

Gene	Mean start	Mean end	Mean bp	Mean % difference
ND1	2840	3814	975	1.95
ND2	4027	5076	1050	2.04
COXI	5467	7017	1551	1.20
COXII^a^	7181	7871	691	0.87
ATPase8	7947	8114	168	0.87
ATPase6	8105	8787	683	1.70
COXIII^b^	8788	9572	785	1.80
ND3	9644	9992	349	1.64
ND4L	10,063	10,359	297	1.45
ND4	10,353	11,733	1381	1.76
ND5	11,946	13,784	1839	1.75
ND6	13,781	14,302	522	1.74
Cytb	14,375	15,515	1141	1.39

^a^ Severy Creek, Colorado, Accession Number 179022 was excluded from COXII analysis because of missing data.

^b^ Figure Eight Lake, Utah, Accession Number 90578 was excluded from COXIII analysis because of missing data.

### 3.1 Geographic Distribution of Haplotypes

All 12 Cutthroat Trout mitochondrial haplogroups identified in our maximum likelihood and Bayesian phylogenetic analyses (Figure 2) were clearly associated with specific drainage basins: Coastal Cutthroat Trout (Pacific Coast), Westslope Cutthroat Trout (Columbia, Fraser, Lower Snake, and upper Missouri Rivers), Lahontan Cutthroat Trout (Lahontan Basin), Yellowstone Cutthroat Trout (Snake River), _BR_YCT (Bear River), Bonneville Cutthroat Trout (Bonneville Basin), _GR_CRCT (Green River basin), _UP_CRCT (upper Colorado, Gunnison, and Dolores rivers), _SJ_CRCT (San Juan River), Rio Grande Cutthroat Trout (Rio Grande, Pecos, and Canadian rivers), Greenback Cutthroat Trout (South Platte River) and Yellowfin Cutthroat Trout (Arkansas River). There were several exceptions to this pattern: we identified some Coastal Cutthroat Trout and Westslope Cutthroat Trout samples with Rainbow Trout haplotypes (Appendix S1). Additionally, due to known widespread stocking of _GR_CRCT and _UP_CRCT across Colorado in the late 1800s and early 1900s, many _GR_CRCT and _UP_CRCT haplotypes were recovered across that state outside of their putative native ranges, particularly in the Arkansas and South Platte River Basins (Metcalf et al. 2012; Bestgen et al. 2019; Appendix S1).

## 4 Discussion

Mitogenomes represent an important source of information for inferring phylogenetic relationships in recently‐diverged taxa (i.e., species and subspecies), especially those with distinct geographic ranges and limited hybridization (Zink and Barrowclough 2008; Stokkan et al. 2018). Furthermore, because of their widespread availability, mitogenomes can significantly increase taxonomic breadth in a study, and the inclusion of full mitogenomes can resolve polytomies in studies where partial mitogenomes failed to clarify relationships among taxa including in mammals, crustaceans, amphibians and insects (Metcalf et al. 2012; Loxterman and Keeley 2012; Stokkan et al. 2018; Chan et al. 2022; Jiang et al. 2022; Yu et al. 2025). Finally, because mtDNA can be consistently extracted from degraded museum samples relative to nuclear DNA, mitochondrial genes and genomes may represent the only genetic line of evidence that can be evaluated across all extinct and extant species in phylogenetic studies. We use Cutthroat Trout as a case study, to illustrate the practical strengths mitogenomes can provide towards species delimitation when taxonomic relationships are disputed, as well as inform conservation efforts, management plans and ESA‐related decisions.

Using full mitogenomes, we identified 12 mitochondrial haplogroups, which consistently correlated with distinct basins/regions (Figure 2, Figure S1). Full mitogenomes offered more resolution than previous studies limited to few mitochondrial genes (e. g. Wilson and Turner 2009; Loxterman and Keeley 2012; Metcalf et al. 2012) and provided strong support for the haplogroups. These haplogroups had a moderate level of divergence (1.30%) between Cutthroat Trout supporting the presence of distinct subspecies. Excluding the _BR_YCT—Yellowstone Cutthroat Trout haplogroup, pairwise distances ranged from 0.65% to 1.97%, highlighting a similar level of divergence between lineages (Table 2). These findings align generally with Behnke's phylogenies based on meristic counts, morphometric measurements, coloration, spotting patterns and geographic distribution that recognized 14 subspecies of Cutthroat Trout organized across four ‘major’ subspecies (Behnke 1992, 2002).

**TABLE 2:** Pairwise average genetic distances between broadly divergent monophyletic clades that coincide with accepted subspecies or undescribed lineages of Cutthroat Trout are compared with each other, Rainbow Trout, and Chinook Salmon.

Clade	CS	RB	AT	RT	CCT	WCT	LCT	YCT	_BR_YCT	BCT	_GR_CRCT	YFC	RGC	GBC	_SJ_CRCT	_UP_CRCT
Chinook (CS)	0.000															
Redband (RB)	0.066	0.000														
Apache (AT)	0.066	0.011	0.000													
Rainbow (RT)	0.064	0.010	0.010	0.000												
Coastal (CCT)	0.067	0.049	0.049	0.048	0.000											
Westslope (WCT)	0.067	0.049	0.049	0.047	0.013	0.000										
Lahonton (LCT)	0.069	0.051	0.050	0.049	0.013	0.011	0.000									
Yellowstone (YCT)	0.070	0.050	0.051	0.049	0.015	0.014	0.015	0.000								
Bear River (_BR_YCT)	0.070	0.050	0.050	0.049	0.016	0.015	0.016	0.003	0.000							
Bonneville (BCT)	0.069	0.051	0.051	0.050	0.017	0.016	0.017	0.012	0.012	0.000						
Green River (_GR_CRCT)	0.070	0.051	0.051	0.050	0.016	0.015	0.016	0.010	0.011	0.010	0.000					
Yellowfin (YFC)	0.072	0.053	0.053	0.052	0.020	0.018	0.019	0.014	0.014	0.012	0.011	0.000				
Rio Grande (RGC)	0.071	0.052	0.052	0.051	0.019	0.017	0.018	0.013	0.013	0.012	0.011	0.009	0.000			
Greenback (GBC)	0.070	0.050	0.051	0.050	0.017	0.016	0.016	0.011	0.011	0.010	0.010	0.011	0.010	0.000		
San Juan (_SJ_CRCT)	0.070	0.050	0.051	0.050	0.017	0.016	0.017	0.012	0.012	0.011	0.009	0.011	0.011	0.008	0.000	
Uncompahgre (_UP_CRCT)	0.070	0.050	0.050	0.050	0.016	0.016	0.016	0.011	0.012	0.010	0.008	0.010	0.010	0.007	0.007	0.000

In fact, despite the broad array of molecular tools used to characterize phylogenetic relationships among Cutthroat Trout, the results have been surprisingly consistent with Behnke's proposed taxonomy. Past studies used allozymes (Loudenslager and Gall 1980; Allendorf and Leary 1988; Shiozawa and Williams 1992; Utter and Allendorf 1994), partial mitogenomes (Metcalf et al. 2007, 2012; Pritchard et al. 2009; Wilson and Turner 2009; Loxterman and Keeley 2012; Shiozawa et al. 2018; Bestgen et al. 2019) Y‐chromosome sequences (Brunelli et al. 2013), microsatellites (Metcalf et al. 2007; Pritchard et al. 2007), Amplified Fragment Length Polymorphisms (Metcalf et al. 2007; Bestgen et al. 2019), and nuclear SNPs (Houston et al. 2012), all revealed similar patterns along with some inconsistencies at the subspecies level. Additional studies covering the nuclear genome continue to be produced, providing further resolution. Kokkonen et al. (2024) examined 1827 nuclear genes and found many of the same relationships identified in this study. Clark (2025) also produced consistent phylogenies examining ~16 million SNPs spread across the nuclear genome. Interestingly, that study revealed some discord between what we and others have found in the mtDNA. For example, nuclear DNA from the _SJ_CRCT aligned more closely with _GR_CRCT, while their mitogenomes were most closely related to _UP_CRCT. In addition, the nuclear genome of South Hayden Creek fish (_UP_CRCT east of the Continental Divide in the Arkansas River Basin) aligned more closely with Rio Grande Cutthroat Trout, while their mitogenome was clearly _UP_CRCT.

This discord reflects additional unexplained relationships between different Cutthroat Trout evolutionary lineages and highlights the need for additional work to completely resolve the convoluted taxonomy of Cutthroat Trout. Mito nuclear discordance is well documented across diverse animal groups, with numerous studies demonstrating that mitochondrial genealogies can conflict with nuclear species trees due to introgression, incomplete lineage sorting, or selection (e.g., Funk and Omland 2003; Toews and Brelsford 2012; Good et al. 2015). Such cases—including examples from Salmonidae (Dallaire et al. 2025; Crespi and Fulton 2004; Englmaier et al. 2024)—underscore that mtDNA represents only a single, maternally inherited locus and must be interpreted alongside nuclear genomic evidence when drawing taxonomic conclusions. Never‐the‐less, much can be gleaned from the full mitogenome phylogenies presented here—particularly with regard to some subspecies‐specific findings that warrant further discussion:

### 4.1 Coastal Cutthroat Trout

Behnke (2002) hypothesized that the ancestor of all Cutthroat Trout lived in a marine environment, and this is supported by the Coastal Cutthroat Trout as being the basal lineage using partial mitogenomes (Metcalf et al. 2007, 2012; Pritchard et al. 2009; Wilson and Turner 2009; Loxterman and Keeley 2012; Shiozawa et al. 2018; Bestgen et al. 2019). Despite this, different data types, including allozymes and nuclear transcriptomes, have identified the Westslope Cutthroat Trout as being basal to all other subspecies of Cutthroat Trout (Loudenslager and Gall 1980; Allendorf and Leary 1988; Shiozawa and Williams 1992; Utter and Allendorf 1994; Kokkonen et al. 2024). With the allozyme and nuclear DNA phylogeny, natural Westslope Cutthroat Trout introgression with Rainbow Trout could also explain why phylogenetic analyses have placed the Westslope Cutthroat Trout as the most basal lineage. For example, Kokkonen et al. (2024) identified a Westslope Cutthroat Trout collected in Idaho's Middle Fork Salmon River as the most basal Cutthroat Trout lineage. This could be explained by previous work that has found widespread Rainbow Trout–Westslope Cutthroat Trout hybridization in the Salmon River System (Kozfkay et al. 2007). Although our samples were limited for Westslope cutthroat trout, we identified two fish from the John Day River drainage that had Rainbow Trout mitogenomes. While mtDNA cannot detect hybridization within an individual, the presence of mixed haplotypes in a population indicates the potential for intermixing. This confirms that Westslope Cutthroat Trout‐Rainbow Trout introgression exists in at least the John Day River System. The high bootstrap (100) in our mphylogenies indicate that Coastal Cutthroat Trout is the most basal Cutthroat Trout lineage, and the misidentified John Day River Westslope Cutthroat Trout haplotypes suggest introgression may at least partially explain conflicting results from other studies.

### 4.2 Lahontan Cutthroat Trout

One mitogenome of particular interest came from Guano Creek in Oregon. Guano Creek historically had a native Redband Trout (*O. m. ssp*.) population. However multiple lineages of Cutthroat Trout, including Alvord Cutthroat Trout (*O. c. alvordensis*), Willow‐Whitehorse Lahontan Cutthroat Trout and Heenan Lake Lahontan Cutthroat Trout (Behnke 2007) were stocked into this system. Thus, we expect that if a fish in Guano Creek had a Lahontan Cutthroat Trout mitogenome, it would be similar to the mitogenomes found in one of these three lineages. We found that the Guano Creek mitogenome was identical to the haplotype found in Mahogany Creek, which drains into Summit Lake in Nevada. Mahogany Creek is a hypothesized entry point for Lahontan Cutthroat Trout into the Alvord Basin via a headwater transfer (Curry and Melhorn 1990; Behnke 2002). Thus, this mitogenome may have originated from the extinct Alvord Cutthroat Trout. Additional comparisons made to a partial mitogenome recovered from a known formalin‐fixed Alvord Cutthroat Trout also found a close relationship between the Alvord Cutthroat Trout and Summit Lake Lahontan Cutthroat Trout (Mussmann 2024). More comparisons between formalin‐fixed Alvord Cutthroat Trout and Lahontan Cutthroat Trout DNA are needed to confirm this hypothesis.

### 4.3 Bonneville and Yellowstone Cutthroat Trout

One needed revision to the taxonomy of Cutthroat Trout is the grouping of native trout in the Bear River Basin with Bonneville Cutthroat Trout (*O. c. utah*) from the southern (main) part of the Bonneville Basin. Behnke hypothesized that the Cutthroat Trout in the Bear River gave rise to the Bonneville Cutthroat Trout when the Bear River was diverted into the Bonneville Basin approximately 50,000 years ago (Pederson et al. 2016). This is consistent with his hypothesis that Cutthroat Trout diversified ~1–2 Ma (Behnke 1992, 2002). However, fossil evidence suggests that divergence between Rainbow and Cutthroat Trout had occurred by 10.2 Ma (Stearley and Smith 2016), highlighting that the split occurred earlier than the 2 Ma Behnke (2002) proposed. Further, numerous genetic studies based on allozymes, mtDNA and nuclear genes, have found that the native trout in the Bear River are sister to Yellowstone Cutthroat Trout, not the Bonneville Cutthroat Trout (Houston et al. 2012; Kokkonen et al. 2024; Loxterman and Keeley 2012; Martin et al. 1985; Shiozawa and Evans 1995; Toline et al. 1999). Our results support Cutthroat Trout in the Bear River being considered a distinct lineage most closely allied with the Yellowstone Cutthroat Trout.

We highlight this relationship in our phylogenies by treating the _BR_YCT as a discrete lineage sister to the Yellowstone Cutthroat Trout which illustrates its divergence from the main basin Bonneville Cutthroat Trout. While the recent changes to Cutthroat Trout taxonomy have grouped Yellowstone and Bonneville Cutthroat Trout under *Oncorhynchus virginalis*, our calibrated phylogeny estimates the split between the Yellowstone and main basin Bonneville Cutthroat Trout at approximately 1.32 Ma, just two hundred seventy thousand years after the Lahontan and Westslope Cutthroat Trout lineages split. This level of observed divergence highlights why _BR_YCT and Bonneville Cutthroat Trout should no longer be managed under the name Bonneville Cutthroat Trout. While Loxterman and Keeley (2012) recommended that the _BR_YCT be called the Bonneville Cutthroat Trout, and those in the Southern (Main Basin) Bonneville Basin be called the Great Basin Cutthroat Trout, the type specimen for the Bonneville Cutthroat Trout is from Utah Lake, in the central part of the Main Bonneville Basin. Because of this, we recommend that Cutthroat Trout found in the Bear River drainage be called either the Bear River Cutthroat Trout or the Bear River lineage of the Yellowstone Cutthroat Trout.

**FIGURE 1:**
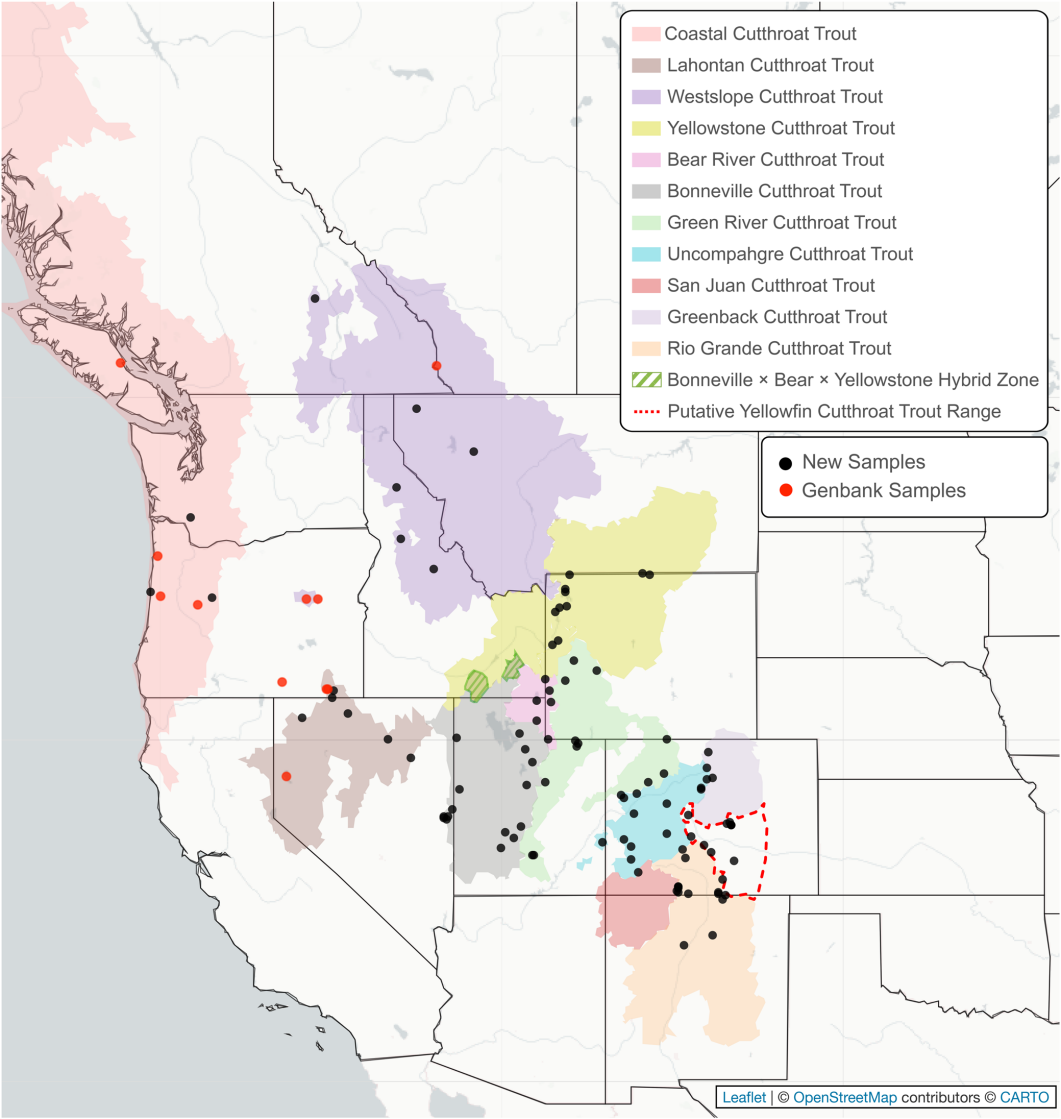
Putative native ranges of Cutthroat Trout (CT) in North America aligned to drainage basins (courtesy of G. Marston, Trout Unlimited). Source locations for new samples sequenced for this study are indicated with black circles, while red ones represent published sequence data from GenBank.

On a finer scale, Yellowstone Cutthroat Trout are parsed into three lineages (Shiozawa and Evans, unpublished data; Campbell et al. 2018): the aforementioned Bear River lineage, a basal Yellowstone Cutthroat Trout lineage in the middle Snake River, and a more recently derived lineage, characteristic of the Yellowstone Cutthroat Trout of the Upper Snake and Yellowstone rivers. Previous studies have identified mixing of mitochondrial haplotypes between these lineages as well as with main basin Bonneville Cutthroat Trout (Campbell et al. 2018). While we did not include any Bonneville Cutthroat Trout samples from the known contact zone in the Middle Snake River (Figure 1), our data further supports the intermixing of the three Yellowstone Cutthroat Trout lineages in the Middle Snake River. Specifically, one sample in the Bear River Basin (Giraffe Creek, Wyoming) had a middle Snake River lineage of the Yellowstone Cutthroat Trout haplotype and another sample in the Middle Snake River Basin (Teton River Tributary, Dog Creek, Idaho) had a _BR_YCT haplotype. These examples could be the result of connections between the Bear River and Snake River basins around the time of the Bonneville flood 17,500 (Benson et al. 2011) to 18,000 years ago (Oviatt 2020). Post flood interbasin connections continued until the final recession of Lake Bonneville below the Provo level, between 15,200 years ago (Benson et al. 2011) and 13,800 years ago (Godsey et al. 2005) allowing for transfer between these basins. We also identified one sample in Woodruff Reservoir (Bear River Basin, Utah) that had an Upper Snake River lineage haplotype. Yellowstone Cutthroat Trout derived from wild spawn operations in Yellowstone National Park (Upper Snake River) were widely stocked across the Western United States (Varley 1979, 1980). This sample likely originated from the large egg‐take operation in Yellowstone National Park (Varley 1979; Varley and Gresswell 1988) and not from natural events. As mitochondrial genomes do not provide the needed resolution to resolve the natural mixing that occurred in the Middle Snake River, more genetic analyses such as genome‐wide marker sampling using next‐generation‐sequencing are needed to understand the distribution of native haplotypes within the Snake River. Museum specimens collected prior to the advent of large‐scale stocking could aid in this discovery if available (e.g., Metcalf et al. 2012).

### 4.4 Cutthroat Trout of the Southern Rocky Mountains

The Southern Rocky Mountain (SRM) region historically included the Colorado River Cutthroat Trout, Greenback Cutthroat Trout and Rio Grande Cutthroat Trout, along with the extinct Yellowfin Cutthroat Trout (Behnke 1992). Mining and other anthropogenic pressures in the late 1800s resulted in native trout populations being over‐exploited or eliminated, especially on the east slope of the Rocky Mountains. By the early 1900s, introductions of non‐native trout across their range caused more permanent declines to native trout populations (Adams 1999; Behnke 1992; McHugh and Budy 2006; Peterson et al. 2004, 2008; Shepard et al. 2002; Shepard 2004). Ultimately, these introductions led to both the Yellowfin and Greenback Cutthroat Trout being declared extinct in the early 1900s (Behnke 2002). However, using mostly meristic characters, Behnke (1992) determined that Greenback Cutthroat Trout historically native to the South Platte and Arkansas River Basins were still represented by a few scattered populations in both river basins. A decades‐long conservation effort then used these fish to repopulate the headwaters of the South Platte and Arkansas basins with the goal of removing Greenback Cutthroat Trout from the federally threatened species list (AMEC 2014). However, work by Metcalf et al. (2007, 2012) determined that most of these relict ‘Greenback Cutthroat Trout’ populations were in fact lineages of Colorado River Cutthroat Trout. By the early 1900s, native trout from Trappers Lake in the headwaters of the White River (tributary of the Green River) were stocked widely across Colorado (Rogers, Bestgen, et al. 2018). In addition, fertilized Cutthroat Trout eggs were taken from the Grand Mesa in the headwaters of the Gunnison River basin in the late 1800s, and distributed into barren waters across the state, including the east slope of the Rocky Mountains. This largely undocumented history of translocations led to long‐term taxonomic confusion with extraordinary management and conservation consequences (Metcalf et al. 2007, 2012; Bestgen et al. 2019). Since this discovery, the Greenback Cutthroat Trout recovery effort has been resurrected with the single remaining population native to the South Platte basin (Rogers, Bestgen, et al. 2018). This work also revealed that the Colorado River Cutthroat Trout actually comprise three distinct lineages (Metcalf et al. 2012; Rogers, Bestgen, et al. 2018). These include (1) Green River lineage native to the Green, White and Yampa river systems along with the Dirty Devil and Escalante rivers in Utah (formerly ‘blue’ lineage *sensu* Metcalf et al. 2012; Rogers, Bestgen, et al. 2018; Bestgen et al. 2019); (2) Uncompahgre lineage native to the upper Colorado, Gunnison and Dolores river basins (formerly ‘green’ lineage *sensu* Metcalf et al. 2012; Rogers, Bestgen, et al. 2018; Bestgen et al. 2019); and (3) the San Juan lineage native to its namesake basin (Rogers, White, and Japhet 2018). These new common names reflect the will of the Colorado River Cutthroat Trout Conservation Team charged with managing all three of these taxa (Rogers 2025).

**FIGURE 2:**
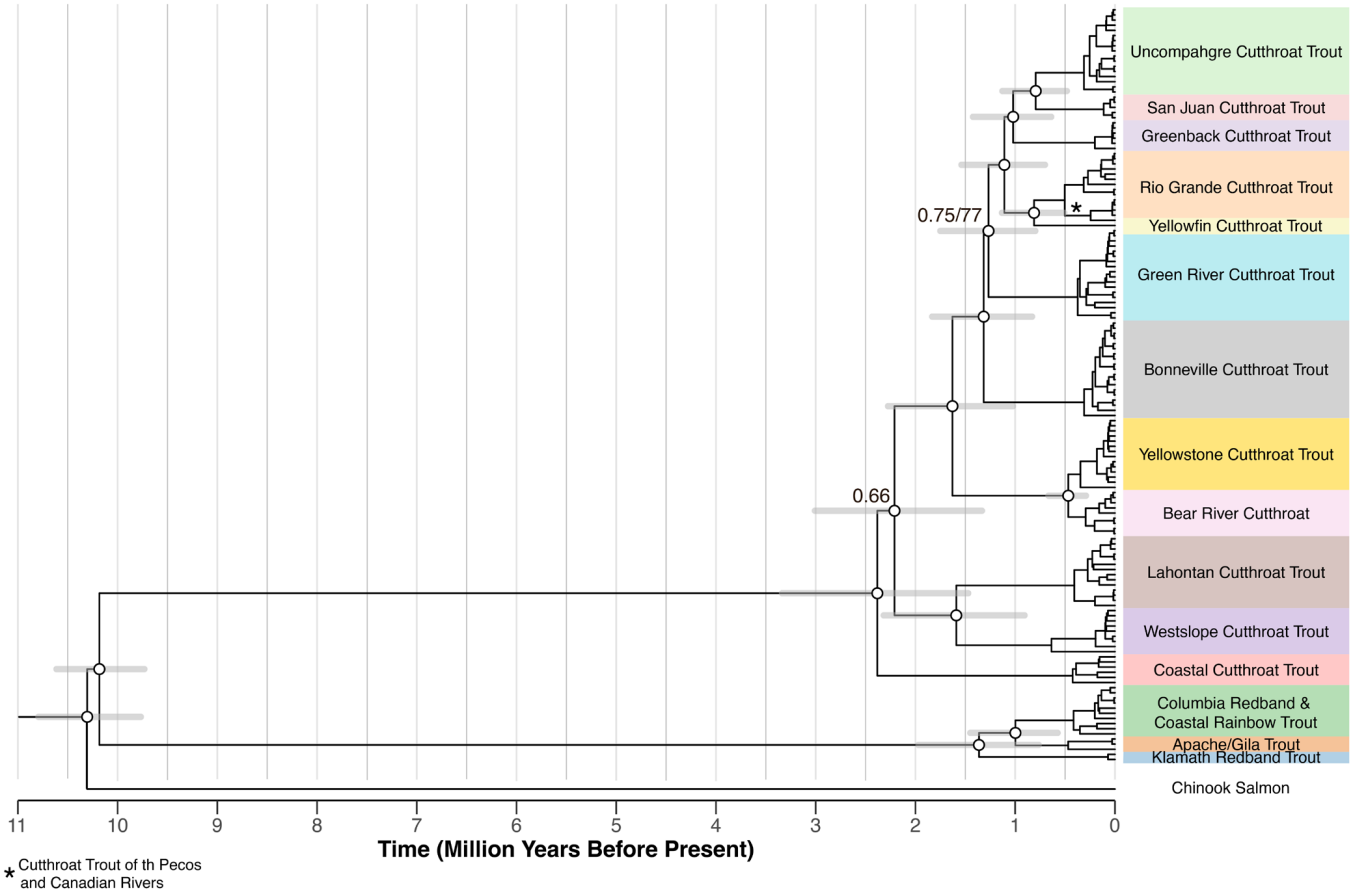
Phylogeny of Cutthroat Trout and Rainbow Trout inferred using Bayesian and maximum likelihood methods, which recovered identical topologies. Time calibration was assessed using Bayesian methods, and the scale bar represented time in millions of years before the present. Node support values are shown as maximum likelihood bootstrap values (*n* = 1000) and Bayesian posterior probabilities for all nodes with a bootstrap value below 90 or posterior probabilities below 0.99.

Despite this complicated history of stocking, over‐exploitation, non‐native invasions and taxonomic confusion, samples in this study reflect all of the major groups of Cutthroat Trout that would have been historically found in the SRM. Multiple ice ages have occurred since Cutthroat Trout invaded this region, leading to cyclic range expansions and contractions, eventually giving rise to the six Cutthroat Trout haplogroups we see today (Figure 2). As such, we recognize the Green River Cutthroat Trout, Uncompahgre Cutthroat Trout, San Juan Cutthroat Trout, Greenback Cutthroat Trout, Yellowfin Cutthroat Trout and the Rio Grande Cutthroat Trout as distinct lineages worthy of conservation. Additionally, while not as distinct as the above lineages, Cutthroat Trout found in upper tributaries of both the Canadian and Pecos Rivers of New Mexico appear to have split from other Rio Grande Cutthroat Trout over 500 ka. Overall, the great diversity of Cutthroat Trout found in the SRM region highlights an ancient and complex evolutionary past.

### 4.5 Major vs. Minor Species Conflict

One of the most intractable challenges in taxonomy hinges on determining an appropriate threshold for delineating species, subspecies and lineages. While seemingly academic, this problem has created many difficulties for species conservation and management. Inconsistent application across species (Mayden 1999) has compounded the problem. Different taxa have varying characteristics that make determining this threshold difficult, including genetically distinct species with overlapping ranges (Cooke et al. 2012; Kautt et al. 2020; Lescroart et al. 2023; Stuart et al. 2006), morphological but not genetic differentiation (Cole et al. 2008; Miller and Benzie 1997), genetic but not morphological differentiation (Cooke et al. 2012; Petzold and Hassanin 2020; Stuart et al. 2006; Xue et al. 2021), and large geographic ranges with variable morphology (Joshi et al. 2023; Mutanen et al. 2012). Cutthroat Trout exhibit many of these confounding issues, including a large geographic range, lineages with morphological variation with little genetic differentiation (Lahontan and Paiute Cutthroat Trout; Snake River and Yellowstone Cutthroat Trout), apparent sympatric ranges with genetic differentiation (Yellowfin and _UP_CRCT), and lineages with subtle morphological differences but substantial molecular ones (_UP_CRCT and _GR_CRCT). In part, these reasons precipitated the need to consider revising the taxonomy, leading to a special workshop at the 2015 annual meeting of the American Fisheries Society to address the issue.

This workshop ultimately decided that the Unified Species Concept (De Queiroz 2007) was a suitable species concept for Cutthroat Trout because of the ability to make taxonomic designations based on the best available data, while also allowing hypotheses to be strengthened and changed over time in response to newly collected evidence. The Unified Species Concept was created to address the century‐long lack of consensus among biologists on what constitutes a species. Many of the species concepts that exist contradict each other and make species designation both more complicated and less consistent. The Unified Species Concept states that a species is a ‘separately evolving’ metapopulation. While reproductive isolation, diagnosability, distinct ecologies, or monophyly can be evidence of differing evolutionary trajectories between metapopulations, these properties are no longer required. De Queiroz (2007) also makes it clear that the lack of any one property (reproductive isolation, diagnosability, distinct ecologies, monophyly, etc.) does not act as evidence contradicting a hypothesis of separate evolutionary trajectories, while emphasizing that any additional evidence only strengthens the hypothesis. By laying out the lines of evidence that distinguish groups of organisms, the Unified Species Concept forces a cataloguing of differences that practitioners can then use to evaluate if full species status is warranted.

In the special workshop, Trotter et al. (2018) proposed elevating the four major lineages described by Behnke as major subspecies to full species. This argument was largely based on geographic, ecological and morphometric data supporting the existence of these four groups, and perhaps a way to honour Behnke's legacy since ‘major’ subspecies are not recognized by the International Commission of Zoological Nomenclature. The four ‘major’ subspecies described by Behnke were based on the assumption that Coastal, Westslope, Lahontan and Yellowstone Cutthroat Trout split from each other one million years ago, with ‘minor’ subspecies arising over the last 100,000 years (Behnke 2002). Our calibrated phylogeny revises these dates, and our pairwise comparisons highlight a much deeper evolutionary history within Cutthroat Trout (Figure 2).

Our phylogeny and pairwise comparisons of genetic divergence (Table 2) do not suggest Coastal, Westslope, Lahontan and Yellowstone Cutthroat Trout are substantially more distinct than at least seven other lineages including the Bonneville, Green River, Uncompahgre, Greenback, Rio Grande, Yellowfin and San Juan Cutthroat Trout. For example, the pairwise distance we observed between Yellowfin Cutthroat Trout, a ‘minor subspecies’ according to Behnke (2002), and Yellowstone Cutthroat Trout is 1.42%. This is on par with differences observed between Yellowstone Cutthroat Trout and other ‘major subspecies’, Lahontan, Westslope and Coastal Cutthroat Trout (1.55%, 1.40% and 1.53% respectively). Similarly, the Rio Grande Cutthroat Trout was also considered a minor subspecies by Behnke (2002), yet its observed pairwise distance with Yellowstone Cutthroat Trout was 1.33%, very similar to the other ‘major subspecies’. Further, we see large divergence between Rainbow Trout and Cutthroat Trout (species, 4.94%), and much smaller differences between these 12 lineages of Cutthroat Trout (1.30%)—consistent with the long‐held practice of referring to them as subspecies. In addition to the support in our study, many previous studies have found these lineages consistently monophyletic across both mitochondrial and nuclear genomes (Houston et al. 2012; Kokkonen et al. 2024; Metcalf et al. 2007, 2012; Rogers, Bestgen, et al. 2018; Shiozawa et al. 2018) and morphologically distinct (Behnke 2002, 1992; Bestgen et al. 2013, 2019). Being both morphologically differentiated and monophyletic are characteristics that support these eleven lineages as being distinct but does not mandate discarding 40 years of precedent. This highlights that the ‘interim classification’ described by Markle (2018) does not accurately describe the diversity found within Cutthroat Trout. As such, we continue to recognize a single Cutthroat Trout species ( *Oncorhynchus clarkii* ) here, comprising eleven distinct groups that likely deserve subspecies designation (with Bear River Cutthroat Trout as a less divergent lineage of Yellowstone Cutthroat Trout; Table 3). This arrangement acknowledges the profound diversity within Cutthroat Trout, facilitates consistency with existing conservation efforts, management plans, and ESA‐related decisions, all while preserving stability associated with historic nomenclature. We acknowledge that continued research into the nuclear genome of Cutthroat Trout may provide evidence to further refine the taxonomic relationships between distinct lineages. Ultimately, taxonomic changes should only follow if existing nomenclature is proven inaccurate, rather than altered based on personal opinions of what should constitute species‐level thresholds.

**TABLE 3:** Mitochondrially distinct Cutthroat Trout lineages, including status assigned by the U.S. Fish and Wildlife Service (USFWS), Committee on the Status of Endangered Wildlife in Canada (COSEWIC) or other sources.

Common name	Scientific name	Current status	Source
Coastal Cutthroat Trout	*O. c. clarkii*	Not at risk of extinction, though some populations are decreasing	Behnke (2002), Pearcy et al. (2018)
Westslope Cutthroat Trout	*O. c. lewisi*	Evaluated for listing under the U.S. Endangered Species Act in August 2003 and found to not be warranted. Listed as threatened in Alberta and special concern in British Columbia SARA	COSEWIC (Committee on the Status of Endangered Wildlife in Canada) (2006), USFWS (U.S. Fish and Wildlife Service) (2003)
Lahontan Cutthroat Trout	*O. c. henshawi*	Threatened, U.S. Endangered Species Act	USFWS (U.S. Fish and Wildlife Service) (2009)
Yellowstone Cutthroat Trout	*O. c. bouvieri*	Evaluated for listing under the U.S. Endangered Species Act in February 2006 and found to not be warranted	USFWS (U.S. Fish and Wildlife Service) (2006)
Bonneville Cutthroat Trout	*O. c. utah*	Evaluated for listing as under the U.S. Endangered Species Act in September 2008 and found to not be warranted. Bear River Cutthroat Trout were included with Bonneville Cutthroat Trout in this ruling	USFWS (U.S. Fish and Wildlife Service) (2008)
Green River Cutthroat Trout	*O. c. ssp*.	Colorado River Cutthroat Trout were evaluated for listing as under the U.S. Endangered Species Act in June 2007 and found to not be warranted. Green River Cutthroat Trout were considered part of the broader Colorado River Cutthroat Trout complex in this ruling	USFWS (U.S. Fish and Wildlife Service) (2007)
Rio Grande Cutthroat Trout	*O. c. virginalis*	Evaluated for listing under the U.S. Endangered Species Act in 2014 and December 2024 and found to not be warranted	USFWS (U.S. Fish and Wildlife Service) (2014), USFWS (U.S. Fish and Wildlife Service) (2024)
Yellowfin Cutthroat Trout	*O. c. macdonaldi*	Extinct	Juday (1906), Behnke (2002)
Greenback Cutthroat Trout	*O. c. ssp*.	Threatened, U.S. Endangered Species Act	Metcalf et al. (2007, 2012), USFWS (U.S. Fish and Wildlife Service) (1998)
San Juan Cutthroat Trout	*O. c. ssp*.	Discovered in 2012, this lineage of Colorado River Cutthroat Trout was considered extinct until 2018 when a half dozen populations were identified	Metcalf et al. (2012), Rogers, White, and Japhet (2018)
Uncompahgre Cutthroat Trout	*O. c. ssp*.	Colorado River Cutthroat Trout were evaluated for listing as under the U.S. Endangered Species Act in June 2007 and found to not be warranted. Uncompahgre Cutthroat Trout were considered part of the broader Colorado River Cutthroat Trout complex in this ruling	USFWS (U.S. Fish and Wildlife Service) (2007)

*Note:* Modified from Penaluna et al. (2016).

### 4.6 Future Directions With Mitogenomes

Although mitogenomes only represent a single, maternally inherited locus and thus a single gene tree, mitogenomes still represent an important line of evidence for documenting relationships in species. Thus, the approach and findings of this study are not limited to Cutthroat Trout. Many taxa such as mammals, crustaceans, amphibians and insects exhibit the same challenges we face delineating Cutthroat Trout subspecies (Yu et al. 2025; Stokkan et al. 2018; Jiang et al. 2022; Chan et al. 2022). This study highlights the utility of using mitogenomes to investigate species delimitation and taxonomy, especially in recently diverged, geographically isolated taxa that are morphologically similar. Further, with the advent of museumomics and the increased ability to sequence genetic markers from historic and ancient samples, this approach can provide novel insight on relationships that were previously defined based on morphological data or limited genetic data (de Abreu‐Jr et al. 2020; Delling et al. 2023). These data can increase taxonomic representation, reveal significant diversity within taxa, and can necessitate major taxonomic revisions (de Abreu‐Jr et al. 2020). Thus, the consistent nature of mitogenomes across the tree of life means mitogenomes will continue to be a valuable point of comparison for different species complexes. As stated above, these principles can be applied to any species complex with contentious taxonomic boundaries to identify and protect distinct lineages as well as create management plans, define conservation units, and aid ESA‐related decisions.

## 5 Conclusion

Understanding the evolution of an organism isn't achieved in isolation, but instead through combining the study of biogeography, morphology, as well as molecular methods. Using just morphology and geography, Behnke (2002) identified evolutionary patterns in Cutthroat Trout that provided a framework for later molecular investigations. Likewise, our work builds on that scaffold using full mitochondrial genomes to include additional insight and resolution over earlier studies that examined only small portions of the mitogenome. While acknowledging the shortcomings of mitochondrial DNA work, we hope that this phylogeny ushers in the final chapter of the perspective mitochondrial DNA can provide towards resolving the complicated evolution of Cutthroat Trout. We hope it serves as a framework for future molecular work on Cutthroat Trout now that next generation sequencing has made thousands of nuclear loci readily accessible.

Importantly, our results demonstrate that complete mitochondrial genomes can provide meaningful insights beyond what is obtained by only analyzing a smaller subset of mitochondrial genes. Cutthroat Trout are a highly diverse and complex species, with divisions that are pronounced and ancient. The diversification between different Cutthroat Trout lineages started > 2.5 million years ago, with eight lineages of Cutthroat Trout that diverged > 1 million years ago, and at least 12 lineages that diverged > 790 thousand years ago. Long branch lengths between haplogroups highlight their distinctiveness and help provide clarity for subspecies designations that have been contested for decades. Continuing to group all lineages as a single species under *Oncorhynchus clarkii* acknowledges their shared evolutionary history, allows consistency with the past 150 years of taxonomic discovery, and provides a framework for formal redescription of subspecies—critical for facilitating management and conservation activities for the myriad pieces that comprise Cutthroat Trout diversity.

## Funding

This work was supported by Colorado Parks and Wildlife and the Brigham Young University Department of Microbiology and Molecular Biology.

## Conflicts of Interest

The authors declare no conflicts of interest.

## Supporting information


**Appendix S1:** Sample information for all individuals included in this study, including GenBank accession numbers, mitochondrial haplotypes, total mitogenome length, sequencing coverage, sampling location and drainage, sequence origin, special notes and associated previous publications.

**Figure S1:** Phylogeny of Cutthroat Trout and Rainbow Trout inferred using Bayesian and maximum likelihood methods, which recovered identical topologies. Node support values are shown as maximum likelihood bootstrap values (*n* = 1000) and Bayesian posterior probabilities for all nodes between major lineages.

## Data Availability

The code underlying the genetic analyses in this article is available on GitHub at the link [https://github.com/Tsvanorden/Cutthroat_Mitochondrial_Genomes]. All mitochondrial genomes used in this study are publicly available under GenBank BioProject accession number PRJNA1301035.
