# Supplementary material for: Phylogeny of Cutthroat Trout (Oncorhynchus clarkii) Based on Full Mitochondrial Genomes With Comments on Species Delimitation and Taxonomy
Source: Evol Appl. 2026 Jul 15;19(7):e70304. doi: 10.1111/eva.70304 (PMC13373317; doi:10.1111/eva.70304)
Supplement: Supplementary file 2 — Figure S1: Phylogeny of Cutthroat Trout and Rainbow Trout inferred using Bayesian and maximum likelihood methods, which recovered identical topologies. Node support values are shown as maximum likelihood bootstrap values (n = 1000) and Bayesian posterior probabilities for all nodes between major lineages. [file EVA-19-e70304-s001.docx]

Supplementary Figure 1.

Phylogeny of Cutthroat Trout and Rainbow Trout inferred using Bayesian and maximum likelihood methods, which recovered identical topologies. Node support values are shown as maximum likelihood bootstrap values (n = 1000) and Bayesian posterior probabilities for all nodes between major lineages.
